# Cinematic rendering of a burst sagittal suture caused by an occipito-frontal gunshot wound

**DOI:** 10.1007/s12024-021-00387-9

**Published:** 2021-06-09

**Authors:** Dominic Gascho, Michael J. Thali, Rosa M. Martinez, Stephan A. Bolliger

**Affiliations:** Department of Forensic Medicine and Imaging, Institute of Forensic Medicine, University of Zurich, Winterthurerstrasse 190/52, CH-8057 Zurich, Switzerland

**Keywords:** Cinematic rendering, Computed tomography, Magnetic resonance imaging, Gunshot wound, Radiologic wound ballistics, Forensic radiology

## Abstract

The computed tomography (CT) scan of a 19-year-old man who died from an occipito-frontal gunshot wound presented an impressive radiating fracture line where the entire sagittal suture burst due to the high intracranial pressure that arose from a near-contact shot from a 9 mm bullet fired from a Glock 17 pistol. Photorealistic depictions of the radiating fracture lines along the cranial bones were created using three-dimensional reconstruction methods, such as the novel cinematic rendering technique that simulates the propagation and interaction of light when it passes through volumetric data. Since the brain had collapsed, depiction of soft tissue was insufficient on CT images. An additional magnetic resonance imaging (MRI) examination was performed, which enabled the diagnostic assessment of cerebral injuries.

## Case report

The prosecutor commissioned postmortem imaging of a 19-year-old decedent with gunshot injuries to the head. The estimated time between death and evaluation was 13 to 17 h. The decedent presented a slightly star-shaped gunshot wound on the forehead and a gunshot wound at the back of the head underneath his hair, which was approximately twice as large as that on the forehead. The star-shaped wound that was visible on the forehead was indicative of a potential entrance wound; therefore, the wound at the back of the head was considered a potential exit wound. However, a fronto-occipital trajectory did not match the scene where the body was found and where a Glock 17 pistol, a 9 mm bullet, and a cartridge that matched the bullet were collected.

**Fig. 1:**
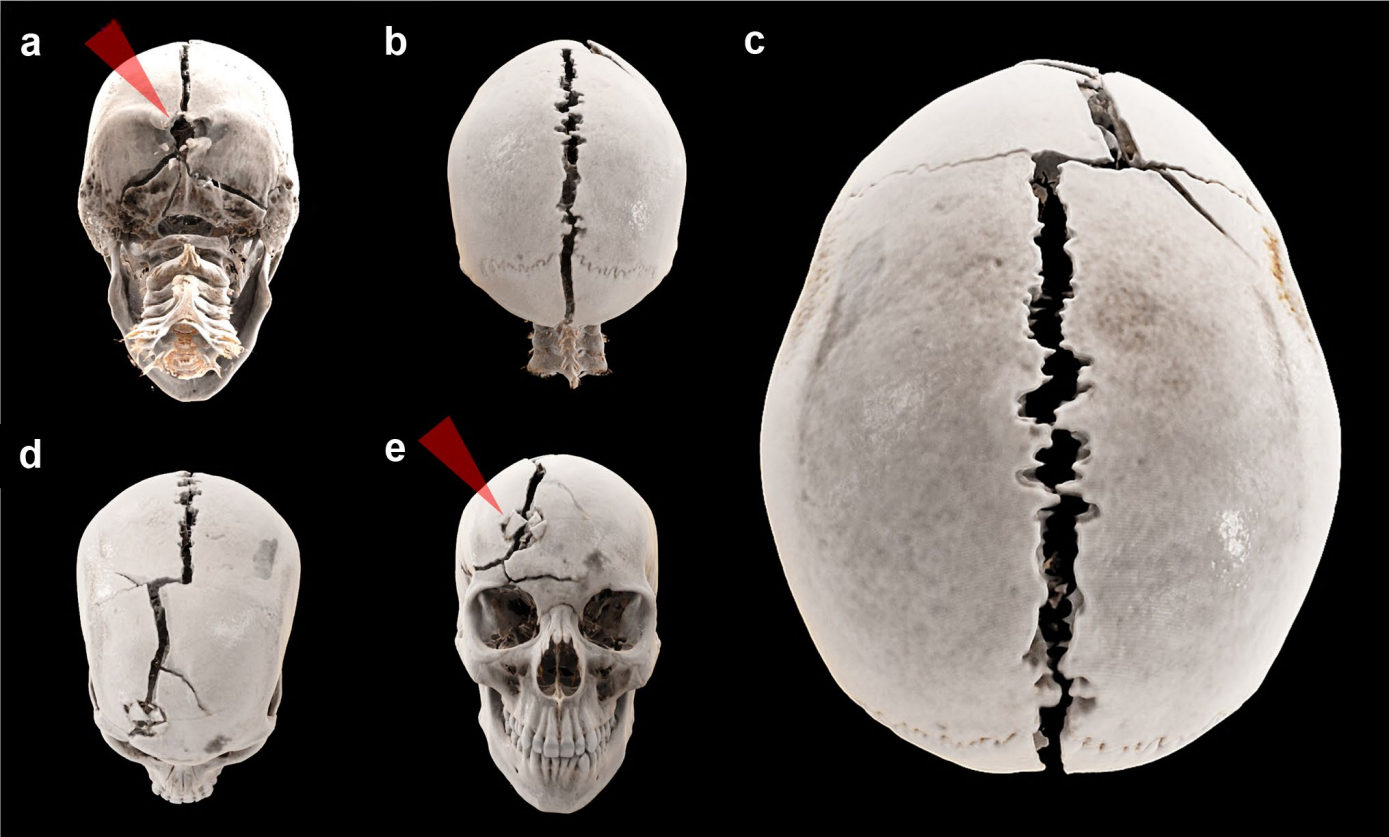
Three-dimensional reconstructions of the skull using cinematic rendering. The occipital bone defect (**a**: arrowhead) presented four radiating fracture lines; of these, the most pronounced line ascended to the external occipital protuberance, where it entered the sagittal suture (**b**). The entire sagittal suture burst (**c**) up to the coronal suture, where a short section of approximately 2 cm of the right-sided coronal suture also burst (**d**). A large fracture line along the frontal bone formed a connecting line between the burst coronal suture and the frontal bone defect (**e**: arrowhead)

**Fig. 2:**
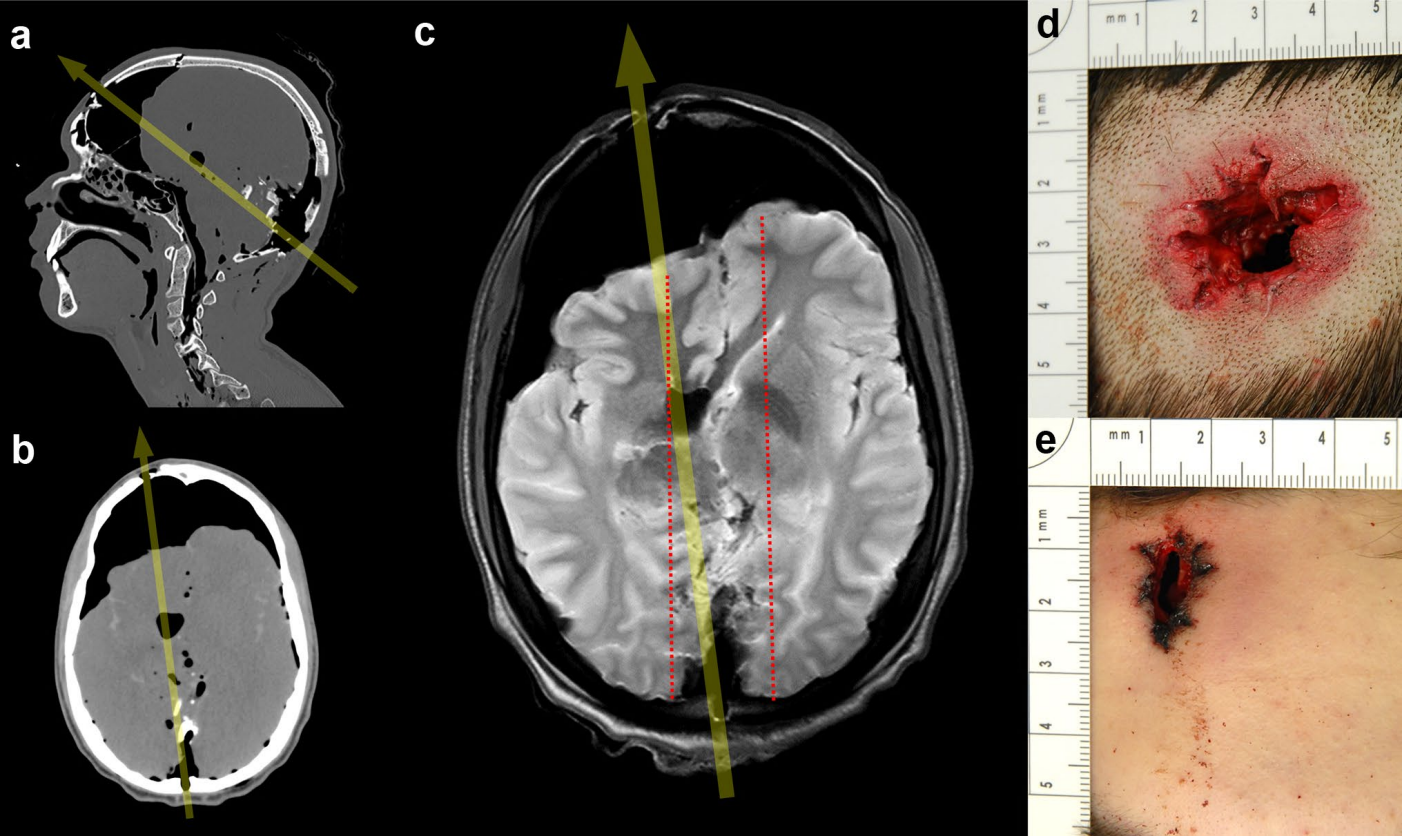
Multiplanar reconstructions of the CT data with a hard kernel (**a**: sagittal view), of the CT data with a soft kernel (**b**: transversal view), of the T2-weighted MRI data (**c**: transversal view), and photographs of the entrance wound, after shaving the hair, (**d**) and of the exit wound (**e**). The bullet entered the occipital bone according to the inwardly shaped bone defect and the bone fragments and exited the head at the frontal bone, resulting in an occipito-frontal trajectory (**a-c**: arrow). On CT images, the bullet path through the cerebral tissue was not depicted and only assumable by air bubbles (**b**), while MRI data clearly delineated soft tissue injuries and the wound channel (**c**: dotted lines)

The decedent underwent a whole body computed tomography (CT) examination using a standard clinical CT scanner (SOMATOM® Definition Flash, Siemens Healthineers, Erlangen, Germany). The scan parameters of the head and neck region were a tube voltage of 120 kVp, a tube current of 600 mAs, and a pitch of 0.35. The reconstruction parameters were a slice thickness of 0.6 mm and a field of view of 250 mm × 250 mm with a soft kernel (H31) and a hard kernel (H60) [1]. Three-dimensional reconstructions were performed using the novel cinematic rendering technique [1–3]. Cinematic rendering of the head presented an impressive fracture line from an occipital bone defect up to a frontal bone defect, where the entire sagittal suture burst (Fig. 1). Multiplanar reconstructions allowed the identification of an occipito-frontal bullet trajectory (Fig. 2a). The occipital bone defect was inwardly beveled; bone fragments in the cerebral tissue were close to this bone defect, though the frontal bone defect was outwardly beveled. The brain had collapsed; thus, CT allowed hardly any anatomical identification of the cerebral structures, and only air bubbles suggested a potential bullet path, as it was assumed that they were distributed along the bullet path (Fig. 2b).

A magnetic resonance imaging (MRI) examination of the head was performed using a standard 3 Tesla MRI unit (Achieva 3.0 TX, Philips Medical System, Best, The Netherlands) and an 8-channel head coil. The MRI protocol included a 4-mm T1-weighted inversion recovery turbo spin echo sequence (repetition time (TR): 2000 ms, echo time (TE): 20 ms), a 4-mm T2-weighted turbo spin echo sequence (TR: 3000 ms, TE: 80 ms), an isotropic T2-weighted turbo spin echo sequence (TR: 2500 ms, TE: 239 ms), and a blood oxygen level dependent (BOLD) sequence (TR: 18.5 ms, TE: 26.2 ms) [4]. MRI delineated the anatomical structures of the cerebral tissue, the bullet path through the cerebral tissue, and some peripheral injuries that were attributed to temporary cavitation (Fig. 2c).

Shaving the back of the head finally revealed a pronounced star-shaped skin defect (size: 3 cm × 2 cm) without visible gunshot residues; the wound margins could not be adapted from this defect. This injury was determined to be the entrance wound of a near-contact shot (Fig. 2d). The slightly star-shaped skin defect with adaptable wound margins on the forehead (size: 0.7 cm × 1.3 cm) was determined to be the exit wound corresponding to the CT findings (Fig. 2e).

## Discussion

Using cinematic rendering, the sagittal suture that burst entirely due to an occipito-frontal gunshot wound was presented in a photorealistic fashion.

Radiating or radial burst fractures of the skull in gunshot wounds were described to be caused by hydraulic pressure that built up in the brain as a result of temporary cavity formation; notably, as the brain is enclosed by a closed and rigid structure, the skull, and high pressure can only be relieved when the skull bursts [5, 6]. With regard to low-energy bullets fired from handguns, long fracture lines that propagate around the entire head are observed in contact and near-contact gunshots, as it is well known that a contact gunshot or near-contact gunshot can cause propellant gas to accompany the bullet into the entrance wound, leading to more severe wounding capacity than intermediate or distant gunshots [6]. In the present case, a near-contact gunshot was determined according to star-shaped wound formation, although gunshot residues were not detected. However, the absence of gunshot residues could be explained by the hair that covered the back of the head. It is conceivable that bursting of the suture was facilitated by the radiating fracture line that directly entered the sagittal suture. Despite the high intracranial pressure in contact or near-contact gunshots and the propagating fracture lines, bursting of a cranial suture due to the high pressure arising from the formation of temporary cavitation seems rare in gunshot victims. Although this finding was already presented on CT images a quarter century ago (in understandably much lower CT image quality) [7], there have not been any more descriptions of this finding in the literature since that time, despite the increasing application of postmortem CT in gunshot victims, aside from a brief mention in a case report [8].

External forces may cause springing (diastasis) of a cranial suture. In infants, it is well known that springing of the sagittal suture can occur after a fall, although there was no additional skull fracture present; in adults, springing of the sagittal suture occurs after falling from a height [9]. Torimitsu et al. [9] applied three-point flexural tests on human bone samples to investigate the biomechanical properties of the sagittal suture in comparison to the parietal bone. The length (L) of the support span was 4·10^-2^ m and the width (w) of the bone sample was 1·10^-2^ m. The thickness (d) of the samples was measured on CT data. Assuming a rectangular cross section, the flexural strength (σ) in pascal (Pa), the stress at failure in bending, can be estimated using the following equation:

$$\sigma =\frac {3 \cdot F \cdot L}{{2 \cdot w \cdot d}^{2}}$$

where F is the force in newton (N) that was required until a fracture occurred, which was measured by Torimitsu et al. [9]. By applying the equation on the measured values of Torimitsu et al. [9] it can be seen that at the middle part of the sagittal suture, σ was less than two times smaller (male samples: 30.3 MPa and 32.6 MPa; female samples: 22.7 MPa and 22.1 MPa) than σ of the left- and right-sided parietal bones (male samples: 65.4 MPa and 71.6 MPa; female samples: 65.0 MPa and 63.9 MPa). At the frontal and occipital parts, the differences in σ decreased between the sagittal suture (male samples frontal: 49.9 MPa, occipital: 41.7 MPa; female samples frontal: 44.5 MPa, occipital: 34.3 MPa) and the parietal bones (male samples frontal: 78.5 MPa and 76.4 MPa, occipital: 68.1 MPa and 69.2 MPa; female samples frontal: 80.8 MPa and 82.5 MPa, occipital: 61.1 MPa and 62.6 MPa).

In addition to hydraulic pressure in gunshot wounds and external force in falls from a height or blunt trauma, diastasis of cranial sutures was also observed postmortem due to freezing [10]; furthermore, burst sutures were described in charred bodies, where thermal pressure can cause cranial sutures to burst [11].

The high pressure that occurred within the skull in the present case is indicated by severe bone defects, broad fracture lines, and collapsed brain tissue. While CT images provide a detailed overview of osseous injuries, they lack information concerning soft tissue injuries. This diagnostic gap was filled by performing an additional MRI examination. MRI allowed for sufficient image quality despite the presence of air in the cerebral tissue. The diagnostic and scientific advantage of MRI over CT for the detection of soft tissue injuries in gunshot victims has been demonstrated in the literature [4, 11–15].
